# Peer-mentees’ challenges in an undergraduate peer-group clinical mentoring programme in a nursing education institution

**DOI:** 10.4102/hsag.v25i0.1435

**Published:** 2020-10-13

**Authors:** Tshepo A. Ntho, Abel J. Pienaar, Leepile A. Sehularo

**Affiliations:** 1 School of Nursing Science, Faculty of Health Sciences, North-West University, Mmabatho, South Africa; 2 Department of Psychology, Faculty of Health Sciences University of Venda, Thohoyandou, South Africa; 3 Department of Graduate Studies and Research, Faculty of Nursing and Midwifery, Shifa College of Nursing, Shifa Tameer-e-Millat University, Islamabad, Pakistan

**Keywords:** Reinforcement, Undergraduate peer-group clinical mentoring programme, Clinical mentoring, Undergraduate nursing student, Clinical learning and teaching

## Abstract

**Background:**

Clinical competency and professional growth amongst nursing students is the cornerstone of a nursing education programme. The demanding and complex training of nursing students requires various clinical teaching and learning strategies such as peer-group clinical mentoring.

**Aim:**

The objective of this research was to explore and describe the challenges that peer-mentees experience in an undergraduate peer-group clinical mentoring programme in one-specific nursing education institution in the North West Province.

**Setting:**

The study was conducted at a nursing education institution in North West Province.

**Methods:**

A qualitative, retrospective case study research design was used. Two separate World Café sessions following a semi-structured schedule based on Gibbs Reflective Cycle were conducted with 51 peer-mentees who were mentored in clinical practice. Four levels of qualitative thematic data analyses were employed to analyse the data.

**Results:**

Five themes emerged from the findings of the study, namely, poor implementation of the peer-group clinical mentoring programme, ineffective undergraduate peer-group clinical mentoring programme, undesirable attitudes of the mentors, mentors unprofessional conduct as well as communication challenges. Ten sub-themes emerged from the findings. Literature control was done to support the findings.

**Conclusion:**

The findings showed that undergraduate nursing students faced a diversity of challenges in the effective learning and teaching of peer-mentees in a clinical context. Limitations and recommendations of the study were given. Recommendations were given for nursing practice, education and research.

## Introduction

Globally, the mentoring programme in nursing education and training has been a subject of discussion for decades and has been recognised as an essential communal relationship (Anderson et al. 2019:2; Seekoe 2013:142). The concept of mentoring is as old as the nursing profession itself (Mariani 2012:2; Olaolorunpo 2019:142). Fallatah et al. (2018:1) defines mentoring as a unique relationship between experienced mentor offering guidance and support to an inexperienced mentee. Bynum (2015:70) adds that mentoring occurs when the more knowledgeable person provides professional support to a less experienced individual specifically designed to nurture both professional and personal growth. Papastavrou et al. (2016:7) and Matlhaba (2016:69) strongly recommend a mentoring programme as an essential clinical teaching and learning strategy that could ensure clinical competency amongst undergraduate nursing students. Yüksel and Bahadır-Yılmaz (2019:52) state that mentoring programme is a beneficial and vital strategy in addressing inadequate academic and social resources that impede the development of professional competence amongst undergraduate nursing students in clinical practice. Subsequently, mentoring amongst undergraduate nursing students is regarded as supportive of a professional programme intended at capacitating undergraduate nursing students with clinical competency and emotional support during clinical learning and teaching (Hale 2018:1; Nersesian et al. 2019:2; Nkutu & Seekoe 2013:51).

Peer-group clinical mentoring programme is an essential and integral strategy for teaching and learning amongst peer-mentees during clinical practice (Fernandez et al. 2018:76). Whilst the benefits of mentoring are well-documented, literature also provides details of the challenges that impede effective clinical mentoring programme (Eller, Lev & Feurer 2014:816). These challenges are also present at Nursing Education Institution (NEI) in North West Province. The challenges include lack of time, workload, lack of support and inadequate formal evaluation of the mentoring programme and stress that burns out the mentors (Eller et al. 2014:816; Fernandez et al. 2018:76; Omeechevarria 2019:47; Wagner 2019:6). In addition, Vuolo (2019:184) states that inadequate provision of mentoring denies undergraduate nursing students an opportunity to learn. The current state of mentorship remains inadequate as it is not properly implemented in many nursing education institutions (Nowell 2019:93; Nowell et al. 2017:2). The rationale of the study is premised on the fact that undergraduate peer-group clinical mentoring is an essential approach in a resource-stricken context to enhance clinical learning and teaching as well as aiding the clinical support and further ensures clinical competence. The realisation that not addressing the mentioned challenges at the specific NEI in the North West Province will perpetuate incompetence amongst undergraduate nursing students, leading to incompetent professional nurses in the clinical setting. The above discussion identifies the gap that there is a need to conduct the research on the challenges experienced by peer-mentees with regard to undergraduate peer-group clinical mentoring programme, particularly at a specific NEI in North West Province where there is literature paucity of research related to this challenge.

## Problem statement

In 2016, one of the NEIs in North West Province introduced the undergraduate peer-group clinical mentoring programme with the aim of improving clinical learning and teaching of undergraduate nursing students and preparing them for clinical practice. Different studies indicate that a mentoring programme for undergraduate nursing students faces challenges despite the desirability for the implementation of various mentoring strategies in NEIs (Beepat 2015:17; Manthata 2016:17; Mlaba & Emmamally 2019:3). These challenges include workload, time commitments, poor communication, lack of support and negative attitudes amongst undergraduate nursing students (Anderson et al. 2019:8; Foster, Ooms & Marks-Maran 2015:19–20). During the researcher’s undergraduate tenure and community nursing service at one of the accredited health establishments, there was evidence that the undergraduate peer-group clinical mentoring programme was dysfunctional and ineffective as a result of various factors such as lack of communication and support within the clinical setting. Motsilanyane (2015:93) reported similar results in the study that explored challenges of clinical orientation in North West Province. From the above information it is clear that there is an inadequate provision of undergraduate peer-group clinical mentoring programmes amongst undergraduate nursing students, especially in a specific NEI in North West Province.

## Research question

What are the challenges that peer-mentees experience on an undergraduate peer-group clinical mentoring programme in a specific nursing education institution in the North West Province?

### Research objective

The research sought to explore and describe challenges that peer-mentees experience on an undergraduate peer-group clinical mentoring programme in a specific nursing education institution in the North West Province.

## Research design

A qualitative retrospective case study as explained by Brink, Van der Walt and Van Rensburg (2012:120,121) was used to gain in-depth understanding of the dynamics in the experiences of undergraduate peer-mentees regarding challenges in the implementation of undergraduate peer-group clinical mentoring programme. Qualitative retrospective case study is a holistic empirical inquiry that looks back at remembered knowledge of a single social unit (Duff 2018:22; Monsen 2018:30; Yin 2014:16).

### Research methods

A qualitative research methods and design was conducted to explore and describe challenges that peer-mentees experience on an undergraduate peer-group clinical mentoring programme in a specific NEI in the North West Province.

### Research setting

There are private and public NEIs in the North West Province. The study was conducted at a public NEI where the students are registered for a bachelor of nursing science undergraduate degree. In this NEI, there is peer-group mentoring programme for the nursing students, which prompted the researcher to conduct this study in the North West Province.

### Population and sampling

The population of this research consisted of all undergraduate peer-mentees registered at a selected NEI in the North West Province. This NEI is accredited by the South African Nursing Council (SANC) to train undergraduate nursing students under Regulation 425 (SANC 1985). There were 92 third years and 56 second years. Purposive or judgemental sampling technique was used to invite 24 undergraduate peer-mentees from the first year and 27 from the second year. In total, 51 undergraduate peer-mentees participated in the study based on their experiences regarding undergraduate peer-group clinical mentoring programme. The sample size of this research was determined by data saturation. The researcher stopped only when there was no new information emerging in the collected data (Hancock et al. 2016:2125). The study included levels two and three undergraduate male and female nursing students who were willing to participate in the study and enrolled for Bachelor of Nursing Science programme at a selected NEI. The study excluded first year nursing students as well as postgraduate nursing students who were registered for masters and doctor of philosophy.

### Data collection

This research used *World Café* as a data collection method following a semi-structured schedule based on Gibbs’ Reflective Cycle (1988). The *World Café* method is a powerful conversational process and useful method of facilitating discussion that assisted undergraduate peer-mentees to re-examine and describe their experiences regarding challenges in the implementation of an undergraduate peer-group clinical mentoring programme. An independent person assisted in the recruitment of participants in September 2019. This was done to avoid researcher bias as he is an alumnus of the university where the data were collected. This study consisted of six groups of participants. In each group, four to five participants discussed a specific principle. Participants divided themselves and chose their own group leaders. This was done to allow the participants to own the discussion, and the researcher was a chief facilitator. The discussion was timed for 15–20 min; and after the time elapsed, the groups rotated and investigated another principle until all groups had discussed all the seven principles. The researcher facilitated two *World Café* sessions with 27 participants and 24 participants per session (51 participants in total) in September 2019. Data were audio recorded and transcribed by the researcher for analysis.

The study followed the six design principles of *World Café* method that Koen et al. (2014:182) describes. These principles were applied as follows. The researcher conducted the study at the nursing school building; participants were the ones who selected this venue because they said it is spacious and will allow productive discussion. The space was friendly and conducive for the undergraduate peer-mentees. The researcher explored relevant topics to be asked to the undergraduate peer-mentees during data collection following the Gibbs’ Reflective Cycle. The researcher encouraged collaborative participation of undergraduate peer-mentees, connecting diverse perspectives of undergraduate peer-mentees. The researcher listened very carefully for emerging patterns and knowledge of the undergraduate peer-mentees; and lastly, he shared collective discoveries with undergraduate nursing students. The discussions lasted approximately 2 hours. The following main question guided the discussions:

1. What are the challenges that peer-mentees experience on an undergraduate peer-group clinical mentoring programme in a specific nursing education institution in North West Province?

### Data analysis

In this research, four levels of qualitative thematic data analysis as described by Pienaar (2017:91) were employed to collate, examine and reflect on these data and generate meanings. This data was analysed as follows:

**Level One:** Concepts were derived from the spoken word of the participants (challenges experienced by peer-mentees of clinical mentoring). The researcher collected and analysed data concurrently with the collaborative participation of participants.

**Level Two:** After building concepts from participants’ experiences regarding challenges experienced by peer-mentees of clinical mentoring, the researcher grouped related concepts into categories with the assistance of participants, and similar categories were themed together to form a logical pattern.

**Level Three:** The researcher, with the close collaboration of participants, identified a new theme during data collection, and this level was followed up through the intuitive deduction of the researcher in which new patterns of data emerged.

**Level Four:** A pattern or storyline was identified, which defined challenges embedded in the implementation of an undergraduate peer-group clinical mentoring programme.

### Measures to ensure trustworthiness

Trustworthiness was ensured through the application of Lincoln and Guba’s criteria of credibility, transferability, dependability and confirmability as cited by Brink et al. (2012:172–173) and Korstjens and Moser (2018:121). Credibility for this research was achieved through the method of data collection (*World Café*), which ensured prolonged engagement in group discussions and clarification from independent leaders of each group. The roles of independent leaders were to allow ownership and proper facilitation of the discussions. Dependability in this research was achieved through sharing collective experiences as suggested and recommended by Koen et al. (2014:182). Confirmability in this research was attained when the findings, conclusions and recommendations followed from the collection of data following *World Café* and extensive notes taken by independent group leaders. Transferability of this research was achieved through the sampling technique in which the sample was selected based on the students’ experiences regarding challenges in the implementation of an undergraduate peer-group clinical mentoring programme at a selected NEI. Detailed description of the research methodology is also given for other researchers who might be interested in understanding how the study was conducted.

## Ethical considerations

Ethical clearance was obtained from North-West University Health Research Ethics Committee (NWU-HREC). Reference Number: NWU-00027-19-A1.

Ethical clearance was obtained from from the director of the School of Nursing Science. Permission to collect data from nursing student mentees was sought from from the director of the School of Nursing Science. Independent person recruited the participants to avoid researcher bias. Informed consent form provided all the information nursing student mentee needed to know before they consented to participate in the research. This information was explained to the student in English as they indicated that it was their preferred language. This was followed by a request to participate in the research at their own volition and the signing of a consent form. Subsequently, participants were informed of their right to refuse to answer any particular question or withdraw from the research at any time.

## Results

Five themes emerged from the findings of the study, namely, poor implementation of the peer-group clinical mentoring programme, ineffective undergraduate peer-group clinical mentoring programme, undesirable attitude of the mentors, mentors’ unprofessional conduct and communication challenges. Table 1 shows the themes and sub-themes that emerged from the findings of the study.

**TABLE 1:** Themes and sub-themes surfaced from the data analysis.

Themes	Sub-themes
Poor implementation of the peer-group clinical mentoring programme	Unstructured and unplanned undergraduate peer-group clinical mentoring programme Lack of commitment and support from nurse educators and practice nurses
Ineffective undergraduate peer-group clinical mentoring programme	Lack of communication Absenteeism of mentors
Undesirable attitude of the mentors	Mentors were unapproachable Disparaging attitude towards undergraduate peer-group clinical mentoring programme Mentors acted as superior and autocratic
Mentors’ unprofessional conduct	Poor time management Some mentors wanted intimate relationships
Communication challenges	Language barrier

### Theme 1: Poor implementation of the peer-group clinical mentoring programme

According to participants, the undergraduate peer-group clinical mentoring programme was poorly implemented at a selected NEI. The following challenges were reported to be hindering the effective implementation of the undergraduate peer-group clinical mentoring programme: the programme was apparently unstructured and unplanned, and there was a lack of commitment and support.

#### Unstructured and unplanned undergraduate peer-group clinical mentoring programme

The undergraduate nursing students spoke about the undergraduate peer-group clinical mentoring programme as not properly established because no guidelines were in place. Nursing educators and NEI management should undertake the evaluation but they have not done so. The following was expressed by participants:

‘[*I*]t was not well established, as there were no guidelines and no monitoring, it was just a word of mouth and it had poor implementation.’ (P7, male, second year, 19 years old)

Another participant said:

‘[*T*]here is poor planning with regard to the peer-group clinical mentoring programme, it was not planned well from the beginning.’ (P24, female, third year, 20 years old)

Another participant added:

‘[*Y*]ou know if something is not well planned, you must expect disaster in the future, so basically, I’m trying to say there was no proper planning with the mentoring programme.’ (P5, male, third year, 20 years old)

#### Lack of commitment and support from nurse educators and practice nurses

Lack of support from nurse educators and nurses at clinical services was reported as having a negative impact on the operationalisation of the undergraduate peer-group clinical mentoring programme at an NEI in North West Province. Participants reported that undergraduate clinical peer-mentors were tasked to do nursing chores without proper induction and support. According to participants:

‘[*I*]t was not done whole-heartedly. It was not well researched and the programme had loopholes.’ (P3, female, third year, 21 years old)

‘[*T*]here is no support from our lecturers or clinic personnel, they just don’t know anything about it.’ (P12, female, second year, 20 years old)

### Theme 2: Ineffective undergraduate peer-group clinical mentoring programme

The second theme related to the unsuccessful implementation of the undergraduate peer-group clinical mentoring programme as experienced by undergraduate nursing student peer-mentees at a selected NEI. Two sub-themes emerged as lack of communication and absenteeism of mentors.

#### Lack of communication

Participants reported no communication between the stakeholders involved (i.e. peer-mentors, peer-mentees and nurse educators). Participants verbalised that mentoring programme was stopped without any proper communication. Some of the participants said:

‘[*C*]linical mentoring programme was introduced only for a month and taken away without informing mentees and we felt like guinea pigs.’ (P6, female, third year, 22 years old)

‘[*T*]hese people did not communicate with us, it just died a natural death.’ (P14, female, second year, 21 years old)

#### Absenteeism of mentors

Participants reported that most of the times some of the mentors absent themselves at clinical services. Some do not even apologise for their absenteeism. This is confirmed below:

‘[*M*]entors were active first day and gave a brief about the clinical setting but they absconded thereafter.’ (P9, female, third year, 20 years old)

Another group echoed similar sentiments:

‘[*T*]hey mentored whenever it suits them and clinical only one mentor was allocated, so when that mentor was absent, there were no mentors.’ (P22, female, third year, 24 years old)

### Theme 3: Undesirable attitude of the mentors

Participants verbalised that mentors were unapproachable, autocratic and displayed undesirable attitudes towards undergraduate nursing students. In essence, the mentors felt superior and this fomented perceptions of negative attitudes towards undergraduate peer-group at an NEI in North West Province.

#### Mentors were unapproachable

According to the participants in this research, most of undergraduate clinical peer-mentors were unapproachable. This had a negative impact on clinical learning and teaching of undergraduate nursing student peer-mentees.

‘[*T*]hey are not approachable, so it was difficult for us to ask questions and to observe other procedures.’ (P20, female, second year, 19 years old)

‘[*Y*]ou know, people are not the same; some people it was not easy to go to them and ask a question, they were so difficult.’ (P16, male, second year, 20 years old)

#### Disparaging attitudes towards undergraduate peer-group clinical mentoring programme

It came out clearly that some of the undergraduate clinical peer-mentors had negative attitudes in mentoring undergraduate nursing student peer-mentees in clinical practice, and these specifically undermined lower level clinical skills. Thus, participants were of the opinion that:

‘[*S*]eniors undermined mentees and are no longer interested in performing clinical skills that were mostly performed at first level.’ (P2, female, second year, 19 years old)

‘[*T*]he big problem with the programme was negative attitude from our seniors, they had attitude, they were very rude to some of my classmates.’ (P18, female, third year, 20 years old)

#### Mentors felt superior and were autocratic

Participants indicated that undergraduate clinical peer-mentors inculcated an autocratic leadership style during clinical learning and teaching as they were ordered to perform social responsibilities of undergraduate clinical peer-mentors. This is confirmed in the following statement:

‘[*M*]entors sent mentees to buy fat cakes for them and they intimidated us.’ (P10, female, second year, 19 years old)

‘[*T*]hey thought they were better than us, and that’s not true, I mean to be in university before me does not mean you are better than me, i don’t know whether you understand what I’m trying to say here.’ (P3, female, third year, 21 years old)

### Theme 4: Mentors’ unprofessional conduct

According to the participants, unprofessional conduct of the undergraduate clinical peer-mentors had a negative impact on their clinical learning and teaching in clinical practices. Poor time management and instances where some mentors wanted intimate relationships with students surfaced as sub-themes.

#### Poor time management

According to participants, undergraduate clinical peer-mentors were late most of the times for clinical practice to facilitate clinical mentoring to undergraduate nursing student peer-mentees. Participants verbalised that little time was spent with undergraduate nursing student peer-mentees in the clinical practice. In addition, they did not have enough time to practise clinical skills demonstrated by undergraduate clinical peer-mentors. Participants spelt out that:

‘[*M*]entors were always late to mentor us in clinics.’ (P5, male, second year, 19 years old)

‘[*T*]here were lot of problems with our mentors, some of them would just come late, and they won’t apologise to anyone, I mean what’s that, you cannot come late and expect other people to come early.’ (P9, female, third year, 20 years old)

#### Some mentors wanted intimate relationships

It is worrisome that the participants in this research spoke out on the unprofessional conduct of undergraduate clinical peer-mentors. According to participants, undergraduate clinical peer-mentors were more interested in having intimate relationships with them rather than mentoring them in clinical practice:

‘[*S*]ome mentors insisted on having personal intimate relationship with mentees.’ (P23, female, second year, 20 years old)

### Theme 5: Communication challenges

Participants in this research stated that communication used to facilitate clinical learning and teaching was one prominent challenge that hindered the effective realisation of the undergraduate peer-group clinical mentoring programme. The language of communication was perceived as a significant barrier.

#### Language barrier

In this research, undergraduate clinical peer-mentors used Setswana to facilitate clinical teaching and learning in clinical practice. Notably, this was a barrier to clinical learning and teaching of other undergraduate nursing student peer-mentees. The following statement confirms this:

‘[*S*]tudents from other provinces felt that the language that was used during mentoring was not a medium for them.’ (P11, female, third year, 20 years old)

‘[*L*]anguage was a problem, especially to some of us, you can’t speak Setswana to me and expect me to follow all the instructions, some of us are not from here, we don’t understand Setswana we.’ (P14, female, third year, 22 years old)

## Discussion

Mentoring is an integral strategy for clinical learning and teaching of undergraduate nursing students in clinical practice, and operational mentoring in clinical practice has great potential to ensure professional development amongst peer-mentees (Chong et al. 2019:2). The findings of this research provided insight into the challenges embedded in the operationalisation of undergraduate peer-group clinical mentoring programme in the clinical practice.

The first theme that emerged was poor implementation. Participants reported ineffectual establishment of the undergraduate peer-group clinical mentoring programme. The poor implementation exacerbated the challenges associated with mentoring programme and resulted in lack of mentoring peer-mentees in the clinical context. In the study conducted by Kibbe et al. (2016:904), it is highlighted that mentorship programmes that are well established have shown significant difference, more specifically in its processes. Henry-Noel et al. (2019:630) refer to informal mentoring as a self-selection of mentors and mentees. In this form of mentoring programme, there is neither a specific clinical learning outcome nor a formal training for stakeholders (Henry-Noel et al. 2019:630). Hence, Gan (2019:6) indicates that the correct implementation of formal mentoring programmes is integral in ensuring that nurses receive mutual clinical support.

According to Nowell (2017:139), the key barrier to facilitating mentorship programme is lack of leadership and direction from management. In the study conducted by Esbenshade et al. (2019:5), lack of commitment to mentorship amongst mentees and mentors was rated very dismally, and this raises serious concern on the efficacy of such an initiative. An examination of the literature suggests that various factors are barriers to successful operationalisation of undergraduate peer-group clinical mentoring programme. These factors include support and commitment (Moores, Holley & Collen 2018:802; Straus et al. 2013:8).

Language barrier in this research emerged as a strong hindrance. South Africa is a multilingual country and higher education institutions register students from all over. However, participants in this research reported that peer-mentors dominantly use *Setswana* and this becomes a barrier to those peer-mentees from other provinces in effective mentoring programme. According to Bock and Schulze (2016:11), language as an important aspect of culture is also fundamental to effective mentoring to ensure that all stakeholders understand what is expected of them. According to Moores et al. (2018:802), inadequate communication bars effective mentoring. Interestingly, in the study conducted by McBride, Campbell and Deming (2019:159), effective mentoring can be facilitated by regular communication between stakeholders. It is evident that good communication skills are important for the effective operationalisation of mentoring (Fokuo et al. 2017:263).

Peer-mentors in this research were reported to be unapproachable and this hindered the effectiveness of the mentoring amongst peer-mentees in clinical practice. This confirms the results of a study by Meyer, Louw and Ernstzen (2017:4) who explored perceptions of physiotherapy students’ regarding the dual role of the clinical educator as mentor and assessor and found that clinical educators in clinical practice were impatient and unapproachable at times. The effective operationalisation of clinical mentoring programme in clinical practice is influenced by various factors that call for approachable and supportive peer-mentors.

Amongst other barriers to the successful operationalisation of the clinical mentoring programme is inculcating an autocratic management style. It came out clearly that peer-mentors in this research were oppressive towards peer-mentees, and this has been reported as another key challenge in effective clinical mentoring programme. Similarly, the research conducted by Donough and van der Heever (2018:5) highlighted the misuse of power entrusted in them by clinical supervisors. Lekalakala-Mokgele and Caka (2015:5) also asserted that in the context of nursing, horizontal violence finds expression in intimidating and bullying other nurses in clinical practice, including misuse of power. Courtney-Pratt et al. (2018:904), describes horizontal violence as abusive behaviours perpetrated amongst nursing students by peer-nursing students. Autocratic behaviour of peer-mentors distracts and demotivates peer-mentees from actively participating in clinical learning and teaching in clinical practice (Minton & Birks 2019:13).

The findings of this study are consistent with the study conducted in the Korle-Bu teaching hospital, which states that time is a challenge for mentees (Oduro-Arhin 2018:86). The aspect of time constraints has been documented as a barrier to effective mentoring (Eller et al. 2014:2; Nowell et al. 2017:9). From the above discussion, it is evident that inadequate time and poor time management are both great challenges to operational mentoring programme amongst peer-mentees in clinical practice.

Peer-mentors are expected to provide and support peer-mentees in clinical settings through the mentoring programme. Mentors are considered as role models who ought to demonstrate high-level professionalism in clinical practice. However, the findings of this research indicated that mentors pursued intimacy relationship with peer-mentees. According to Lin et al. (2018:2), effective mentoring relationships are designed to support and empower mentees to achieve the clinical learning goal. The authors further emphasise that mentoring relationships operate within an ambience of mutual respect that benefits both parties (Lin et al. 2018:2). A mentoring programme broadly rests on the nature of the mentor and mentee’s ethical relationship (Won & Choi 2017:8). Hale (2018:333) states that one of the many responsibilities of a mentor is to clearly define the balance between supporting mentees professionally and being friend.

## Limitations of the study

This research was conducted only in one campus of the NEI in North West Province. This limits the generalisability of the findings to other campuses. However, the findings and recommendations of this research can be applied in other campuses or other NEI in South Africa. Describing the setting and findings in detail would allow other researchers to apply the findings.

## Recommendations

The specific NEI that implements an undergraduate peer-group clinical mentoring programme in North West Province should develop specific guidelines to ensure formal implementation, effective clinical learning and teaching of peer-mentees in the clinical setting. Management and nursing educators of the NEI should support the undergraduate peer-group clinical mentoring programme. There should be collaboration and establishment of clinical practice committee between relevant stakeholders, such as establishing a committee. This committee should comprise of peer-mentees, peer-mentors, nurse educators, registered nurses and management from both health facility and NEI. An ongoing in-service training regarding peer-group clinical mentoring programme for all the involved stakeholders is strongly recommended based on the results of this research. Lastly, further research should be conducted on development of undergraduate peer-group clinical mentoring programme model in the North West Province.

## Conclusion

Despite the benefits of undergraduate peer-group clinical mentoring programme in clinical practice, the research revealed that undergraduate nursing students as mentees face various challenges that need attention for the effective clinical learning and teaching in clinical context. These challenges include poor implementation of mentoring programme, undesirable attitude of undergraduate peer-mentors and unprofessional conduct. Nevertheless, undergraduate peer-group clinical mentoring programme is an essential approach in fostering clinical competency and professionalism amongst nursing students.
